# Study of Optimal Cam Design of Dual-Axle Spring-Loaded Camming Device

**DOI:** 10.3390/ma14081940

**Published:** 2021-04-13

**Authors:** David Rybansky, Martin Sotola, Pavel Marsalek, Zdenek Poruba, Martin Fusek

**Affiliations:** 1 Department of Applied Mechanics, Faculty of Mechanical Engineering, VŠB–Technical University of Ostrava, 17. listopadu 2172/15, 708 00 Ostrava, Czech Republic; david.rybansky@vsb.cz (D.R.); martin.sotola@vsb.cz (M.S.); zdenek.poruba@vsb.cz (Z.P.); martin.fusek@vsb.cz (M.F.); 2 Institute of Thermomechanics of the Czech Academy of Sciences, Dolejskova 5, 182 00 Prague, Czech Republic

**Keywords:** SLCD, friend, cam, topology optimization, FEM, NURBS, MATLAB, SIMP, ANSYS

## Abstract

The spring-loaded camming device (SLCD), also known as “friend”, is a simple mechanism used to ensure the safety of the climber through fall prevention. SLCD consists of two pairs of opposing cams rotating separately, with one (single-axle SLCD) or two (dual-axle SLCD) pins connecting the opposing cams, a stem, connected to the pins, providing the attachment of the climbing rope, springs, which simultaneously push cams to a fully expanded position, and an operating element controlling the cam position. The expansion of cams is thus adaptable to allow insertion or removal of the device into/from a rock crack. While the pins, stem, operating element, and springs can be considered optimal, the (especially internal) shape of the cam allows space for improvement, especially where the weight is concerned. This paper focuses on optimizing the internal shape of the dual-axle SLCD cam from the perspective of the weight/stiffness trade-off. For this purpose, two computational models are designed and multi-step topology optimization (TOP) are performed. From the computational models’ point of view, SLCD is considered symmetric and only one cam is optimized and smoothened using parametric curves. Finally, the load-bearing capacity of the new cam design is analyzed. This work is based on practical industry requirements, and the obtained results will be reflected in a new commercial design of SLCD.

## 1. Introduction

In mountaineering, securing the climber’s safety and preventing falls is of paramount importance. In free climbing, the most common method for fall prevention is the placement of protection gears (PRGs).

### 1.1. Description of Protection Gears

**Figure 1:**
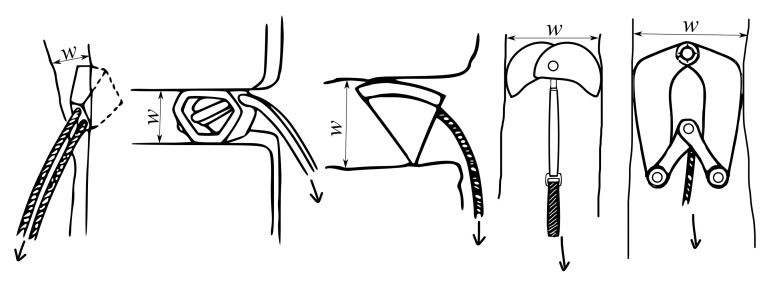
The passive PRG (from left): Nut, Hexentric, Tricam, and the active PRG (from right): LTMS and single-axle SLCD.

The PRGs can be installed permanently (pitons), or temporarily (nuts, hexentrics, tricams, and other devices, see Figure 1, where *w* represents the width of the rock crack and the arrow indicates the direction of the load in the case of a fall [1].

Two basic classes of the PRGs can be distinguished: passive (without moving parts) and active (with moving parts) [2]. Passive PRGs include simple devices able, due to their shape, to exert a force on the rock that is far greater than the primary force acting on the PRG. Such equipment is, therefore, unsuitable for crumbling rocks. Passive PRG devices practically work on the principle of a simple wedge inserted into a converging rock crack into which they can be easily inserted. However, the removal of the device from the rock crack is difficult.

On the other hand, active PRGs typically consist of two pairs of opposing cams that simultaneously expand, thus wedging themselves into the rock crack. The simplest active PRG is the spring-loaded camming device (SLCD), coming in the single-axle and dual-axle variants. They are widely used due to the shape of the cams, which is designed to fit a wide range of cracks. The Lever-Type Mechanical Stopper (LTMS) devices with a more complex mechanism utilizing a flatter shape of cams, patented by our group in 2016 [3] and introduced in the article by Zondlak et al. [4] could be an interesting novel alternative to SLCDs. The cams of active PRGs adapt to allow easy insertion of the device into rock cracks as well as easy removal. Aluminum alloys are widely used materials for the PRGs, offering the advantage of high strength combined with low weight [5].

All PRGs must be tested to ensure safety; in Europe, testing is governed by the EN 12276 standard, which sets the conditions that must be met by the PRG to be allowed on the market. The EN 12276 standard defines testing methods, minimum load-bearing capacity $F_{min}$ = 5.00 kN, testing positions, and other conditions [6].

To compare the capabilities of temporary PRGs with respect to the width of rock crack, working range ratio, a parameter characterizing the breadth of the device usability can be used. It is described by the equation

(1) $$R=\frac{w_{max}-w_{min}}{w_{max}}\cdot 100,$$

where $w_{max}$ and $w_{min}$ represent the maximum resp. minimum width of rock crack (corresponding to cam opening size), see Figure 1.

To compare the individual types of temporary PRGs, data on the extreme working positions of the individual PRGs were obtained from the catalog of a Czech manufacturer, Petr Kouba [7], specializing in the production of climbing equipment. While calculating the ratios, it was noted that the ratios for individual types of PRGs are not constant; the mean values of the individual ratios are presented in Table 1 (rounded to the integer).

The ratio values presented in Table 1 show that, among passive PRGs, tricams have the widest usability range and among the active PRGs, it is the dual-axle SLCD. Furthermore, the dual-axle SLCDs show the best working range ratio of *R* = 41% of all presented temporary PRGs.

### 1.2. Properties of Dual-Axle Spring-Loaded Camming Device

**Figure 2:**
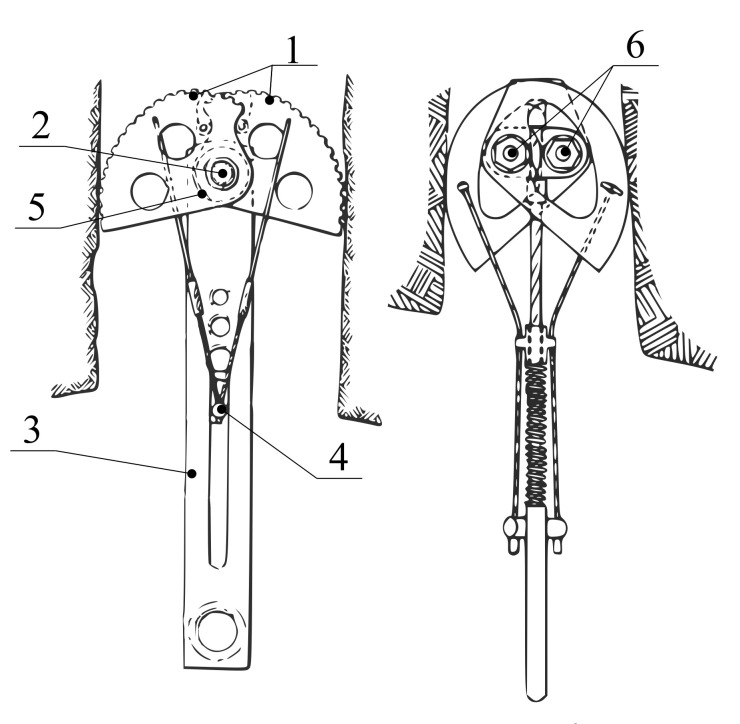
Single-axle SLCD patented by R. D. Jardine (**left**) [8] and dual-axle SLCD patented by T. Christianson (**right**) [9].

SLCD is a simple mechanism utilizing the conversion of the linear movement of the rope into the rotational movement of the cams for a firm attachment into the crack. SLCD in the single-axle variant was introduced in 1980 when it was patented by R. D. Jardine [8], see Figure 2. It consists of two pairs of opposing cams rotating separately (item 1), one pin situated between the opposing cams (item 2), a stem with a loop connected to the pins providing the attachment to the climbing rope (item 3), an operating element which, when pulled, folds the cams into the flattest possible position (item 4) and spring(s), which simultaneously expand the cams (item 5) upon release of the operating element.

Due to their popularity, various active PRGs have been derived from this patent. The currently most widely used variant-Dual-Axle SLCD-was introduced in 1987 by Tony Christianson [9]. It uses two parallel axes (two pins instead of one, see item 6 in Figure 2), which increases the ratio of working range from 35% to 41%, see Table 1. Thanks to its large adaptability, the total amount of equipment (and, in effect, total weight) the climbers need to carry is reduced.

While the pins, springs, looped, and operating elements can be considered optimal, the internal shape of the cams still offers space for improvement. For this reason, this research focused on optimizing the shape of the Dual-axle SLCD cam, which is at present, thanks to its wide working range, the most widely used PRG.

### 1.3. Analysis of the Current Dual-Axle Cam Design

**Figure 3:**
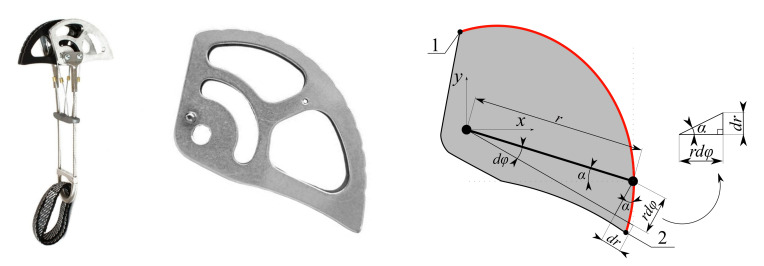
The PB6 SLCD produced by Petr Kouba [7], its cam and a simplified cam model for determination of its contact shape (points on curve: 1—starting point, 2—end point).

The problem can be solved as symmetrical if we accept the following assumptions: (i) The rock crack is vertical and parallel, (ii) the device is inserted ideally (i.e., the loop connected to the pins providing the attachment to the climbing rope is in the vertical direction), (iii) the pin situated between the opposing cams is considered rigid and, lastly, (iv) the friction between the cam and the pin is negligible. Then, the total force acting on the device in the single plane can be decomposed as one-quarter of the force acting upon each cam. This way, it is enough to optimize the shape of a single cam of the device and to apply the same optimized shape to the others. For research purposes, the PERMON BIAXIAL 6 (PB6) cam design was selected as the reference standard (Figure 3). PB6 is designed and manufactured by Petr Kouba company (Tabor, Czech Republic) and is one of their best-selling SLCDs [7]. PB6 declared the minimum cam opening size of $w_{min}$ = 64.0 mm, maximum cam opening size of $w_{max}$ = 108 mm, load-bearing capacity of $F_{max}$ = 14.0 kN, and total weight of $m_{t}$ = 258 g.

The optimal contact shape can be derived using the equation describing the elementary increment of the beam

(2) $$\tan\alpha =\frac{dr}{r\;d\varphi },$$

see a detail of the triangle in Figure 3. Using the Coulomb’s friction model, we can write the slip condition where $f_{T}$ is the minimal coefficient of static friction Using the Coulomb’s friction model, we can write the slip condition where $f_{T}$ is the minimal coefficient of static friction between the rock and the material of the cam preventing the cam from slipping; it can also serve for the calculation of the limiting angle of the beam α. Using the substitution tan α= $f_{T}$ and the separation of variables, we obtain the equation

(3) $$\int_{0}^{\varphi }f_{T}\;d\varphi =\int_{r_{0}}^{r}\frac{1}{r}\;dr\cdot$$

The angle of the beam α below the limit value causes the cam to slip. In the optimal case, the angle α should be constant because a constant value represents uniform loading of the cam in any position. By integrating the Equation (3) and its modification, we obtain the equation of the logarithmic spiral

(4) $$r_{\left(\varphi \right)}=r_{0}e^{f_{T}\varphi }$$

describing the optimal contact shape of the cam [10], see the red curve in Figure 3. The mathematical function describing the shape *r*, therefore, depends on the initial radius of the cam $r_{0}$, the coefficient of static friction between the contact surfaces $f_{T}$, and the rotation of the cam φ in radians. It should be noted that pin friction and other effects of passive resistance are neglected when deriving Equation (4). These effects are, however, reflected in the value of the coefficient of friction $f_{T}$, which is chosen by the designer as a compromise between the magnitude of the coefficient and other parameters, such as the size, weight, material, etc. It should be also noted that, while the theoretical contact curve of the cam is ideally smooth, the manufactured contact surface contains notches that allow superior attachment in a rock crack that weren’t considered in computational models. The optimal contact (external) shape of the cam designed using the Equation (4). has been tested for years under extreme conditions and is verified.

### 1.4. Possibilities of Dual-Axle Cam Design Improvement

The internal shape of the cam, however, may be further improved. Unlike the outer shape, which is more or less designed similarly by all manufacturers, there is no such uniformity in the internal shape and many types are currently on the market. Therefore, the objective of this work was to prepare a method for determining the optimal shape of the cam, facilitating optimal distribution of the material to maximize the design performance with regards to the given conditions of the functionality and load-bearing capacity. The resulting shape should have the best balance of the weight (as low as possible) and stiffness (as high as possible). To meet the objectives of this study, the topology optimization technique using the Finite element method (FEM) was chosen. Preliminary results of the work were published by by the first author of this study in his Master’s thesis [11].

## 2. Materials and Methods

**Figure 4:**
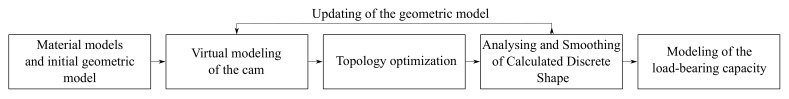
A diagram showing the research workflow of process of obtaining the optimal cam shape.

This chapter will be divided into five subchapters describing the individual stages of the process of obtaining the optimal cam shape in detail. Stage I describes the selection of the material models and the design of the initial geometry model. The next stage (Stage II) presents the virtual modeling of the cam using a linear computational model and its boundary conditions using FEM. Stage III focuses on the topology optimization of the linear computational model. Stage IV presents the analysis and smoothing of the calculated discrete shape. To achieve an even better solution of the geometry, Stages II–IV were performed again on the model arising after the first round of optimization and smoothing. Using this procedure, the ideal cam shape was obtained. In the last stage (Stage V), the load-bearing capacity was modeled using a nonlinear computational model. A diagram showing the research workflow of the process of obtaining the optimal cam shape is depicted in Figure 4.

### 2.1. Stage I: Material Models and Initial Geometry Model

The complex geometry of a cam is easy to be manufactured using a computer numerical control (CNC) machine–mill or laser. For the purposes of this study, the original material of PB6, i.e., the aluminum alloy EN 424254 (AW2011), was chosen. AW2011 uses copper as the alloying element and offers high mechanical strength. The engineering values of material properties of AW2011 are detailed in Table 2; they were obtained from the web [12,13]. The table also contains the yield stress and true value of tangent modulus of the AW2011 material to describe the plastic behavior of the cam for computer modeling of the load-bearing capacity. The true values of tangent modulus and limit plastic strain were calculated using the Yield stress, Ultimate stress, and Elongation of material.

According to the EN 12276 standard, the SLDC’s cam is mounted in parallel steel jaws during laboratory testing. To simulate the experiment and determine the load-bearing capacity of the cam (presented in Stage V), a common material-S235 steel-is used. The material constants [14] are presented in Table 3.

As Equation (4) implies, the coefficient of static friction and initial radius of the cam $r_{0}$ are important parameters determining the optimal contact shape. The coefficient $f_{T}$ differs for various contact pairs of materials. An investigation of the value of the coefficient of friction between aluminum and steel is discussed in a paper by Javadi et al. [15]. Their results show that the coefficient of friction ranges from 0.20 to 0.80, depending on the normal stress. In real conditions, SLCDs are inserted into rock cracks, where the coefficient of static friction is higher. Granitic rocks represent the most common climbing conditions; their coefficient of friction ranges from 0.32 to 0.46 [16]. It is necessary to note that climbing in rainy weather is not expected. Therefore, laboratory testing of SLCD in steel jaws is always on the side of safety. For this reason, a safe value of $f_{T}$ = 0.27 representing the sliding condition between the AW2011 alloy and the S235 steel is considered. It should be noted that the value of $f_{T}$ = 0.27 is the same as used by the manufacturer Petr Kouba for the design of their cams. The limit angle of the beam α then corresponds to α = $\arctan f_{T}$ = 15.1${}^{\circ }$, see Figure 3. The used coefficient of static friction is presented in Table 4.

**Figure 5:**
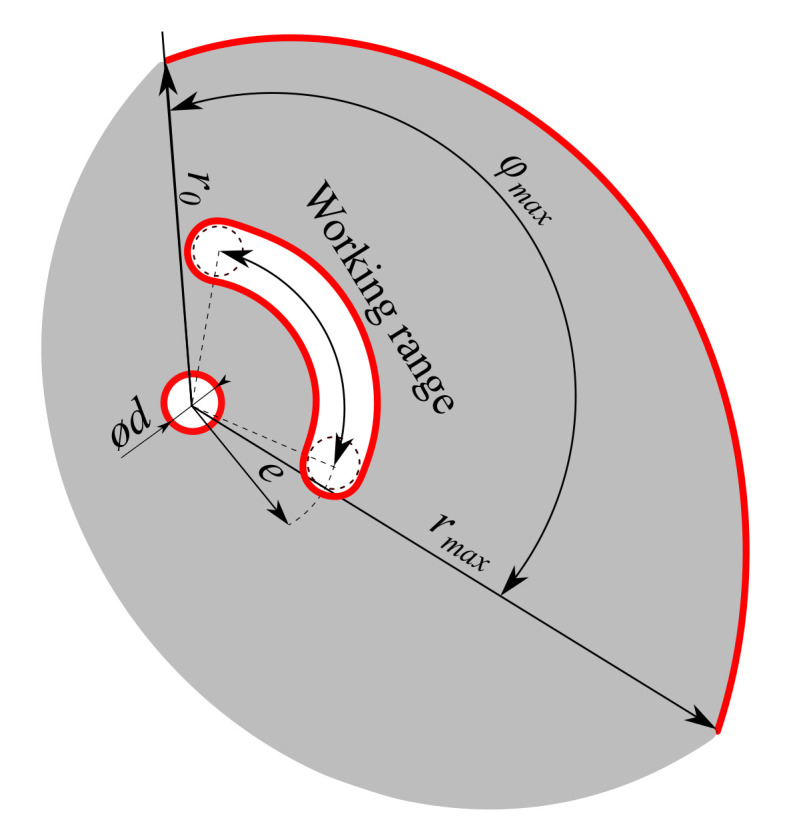
Initial geometric model of the cam.

To create the initial geometric model, the initial radius of the cam $r_{0}$ = 35.7 mm was derived from the PB6’s cam. The optimal contact shape of the cam was generated in the range of the rotation of the cam $\varphi =0^{\circ }$–127${}^{\circ }$ with the uniform step $\varphi_{step}=0.48^{\circ }$ using Equation (4). The maximal radius of the cam $r_{max}$ = 65.0 mm then corresponded to the maximal rotation of the cam $\varphi_{max}=127^{\circ }$. Other initial parameters, such as the thickness of the cam *t* = 6 mm, the diameter of the pin hole ϕd = 6 mm, and the distance between pins *e* = 16 mm were also built on the PB6’s cam to allow improvement of the present cam design. The remaining shape of the initial geometry model was created by complementing the original design with a part of a circle with a large radius to create space for the TOP process (see Figure 5). The parameters required for the construction of the initial geometric model are presented in Figure 5 and Table 5.

Shapes highlighted in red are fixed and cannot be modified during TOP. The initial geometric model was created using the CAD software SpaceClaim Direct Modeler (Ansys, Canonsburg, PA, USA, SCDM).

### 2.2. Stage II: Virtual Modeling of Cam

To design a computational model for solving a structural analysis problem, it is necessary to describe its properties. With regard to the different working positions of the cams, it was necessary to design a multi-load step model that covers all working ranges. A linear elastic behavior (small displacements and strains) was assumed in the range of loading $F_{min}$ = 0–5.00 kN defined by the EN 12276 standard for the whole device. Therefore, the one quarter of the minimum load-bearing capacity $F=F_{min}/4$ = 1.25 kN was considered to represent the loading of each cam. The elastic material properties of AW2011 were used to create a computational model (see Table 2). To simulate real boundary conditions during testing, two types of supports were designed. Support A represents a symmetric boundary condition for the pilot node. Support B simulates the contact condition between the cam and the jaw. Given the complex multi-load step model and following an optimization performed in Stage 2, the procedures using the Finite element method (FEM) were created in MATLAB (MathWorks, Natick, MA, USA) to describe the mechanical behavior of the cam.

**Figure 6:**
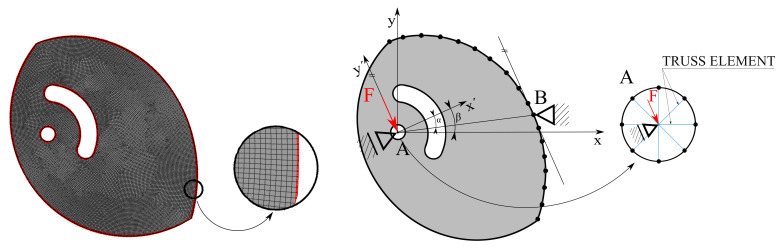
The finite element mesh used for description of the cam (**left**) and boundary conditions of linear computational model (**right**).

A planar computation model of the cam was developed for the analysis and smoothing of the calculated discrete shape in Stage IV. A linear four-node element with plane stress behavior was used for discretization of the initial geometric model. The finite element mesh was generated using ANSYS Workbench (AWB; Ansys, Canonsburg, PA, USA), see Figure 6. The size of the edge of one element was defined as $e_{size}$ = 0.50 mm. The mesh sensitivity analysis was performed for the primary variable of the topology optimization. The proposed discretization does not affect the primary results. A statistics of the mesh is presented in Table 6.

The stiffness matrix of the plane element was calculated using the surface integral

(5) $$\left[k_{plane}\right]=t\int_{S}{\left[B\right]}^{T}\left[C\right]\left[B\right]dS,$$

where the characteristic dimension *t* is the thickness of the cam, [B] represents the strain-displacement matrix, [C] is the stress–strain matrix, and *S* is the surface of the element. The integral is solved numerically. To be able to calculate the primary variable, i.e., the field of displacements, it was necessary to assemble global stiffness matrices. All the element stiffness matrices [*k*] were assembled into a global stiffness matrix [*K*]. The procedures of creating stiffness matrices for used elements can be found in books by Bhatti [17,18]. Similar procedures were also used by Kminek et al. [19]. Further practical advice on the use of FEM in complex applications can be found, e.g., in papers by the group of Horyl and Marsalek [20,21].

Finally, the idealization of the boundary conditions in the presented computational model was another important factor influencing the primary variable. One-dimensional truss elements were used to replace the pin to simplify the model and speed up the proposed procedure, see Figure 6. The stiffness matrix assembly of the truss elements $K_{truss}$ can be found in the book by Bathe et al. [22], and the practical application of truss elements was presented in the article by Klemenc et al. [23]. High Young’s modulus value simulating an almost rigid pin was considered. The global stiffness matrix can be expressed as

(6) $$\left[K\right]=\left[K_{plane}\right]+\left[K_{truss}\right],$$

where $\left[K_{plane}\right]$ is the stiffness matrix of the cam and $\left[K_{truss}\right]$ is the stiffness matrix of truss elements. Because the cam rotates in the working range, the presented multi-load step problem was transformed into separate load cases. Each load-case had a different location of the Support B and orientation of the Support A, which was given by the angle β determining its rotation relative to the global coordinate system XY. The rotation also depends on the size of the coefficient of friction $f_{T}$ (or, respectively, the angle of the beam α), so that the slip condition is maintained, see Figure 6. The number of load-cases depends on the size of the elements; in the presented study, it was equal to 267 load-cases.

### 2.3. Stage III: Topology Optimization

Topology optimization (TOP) is a mathematical method for optimization of the distribution of materials within a structure with respect to boundary conditions. Using this method, it is possible to find a new, more efficient, design obtained through black/white solution [24]. In engineering practice, the first step is to describe the given space using the finite element and density-based methods with SIMP (Solid Isotropic Material with Penalty). Subsequently, the final shape is found using a gradient-based method such as the optimality criteria algorithm. This procedure was presented by Ole et al. [25] including the source code for a plane square element in MATLAB. The procedure of designing the optimal shape using topology optimization was presented by Sotola et al. in their paper, in which the authors described the theory and implementation of the above method using a code in MATLAB [26]. Furthermore, their paper discusses the problem of supplementing the code with the analysis of several load-cases, inserting passive areas, using filtering, etc. Another example of topology optimization procedures has been published by Jancar et al. [27].

The approaches described above were used for topology optimization of a planar problem of the cam. Finite elements were used to discretize the shape of a cam where each element was assigned its dimensionless density $x_{e}$ representing a design variable in topology optimization. This value does not characterize any physical properties of the material; rather, it indicates the importance of the element in the part. In a 2D problem, the design variable can be understood as thickness. The used material model, SIMP, assigns an elasticity modulus $E_{e}$ to each element depending on its density $x_{e}$, according to the equation

(7) $$E_{e}=E_{min}+x_{e}^{p}\left(E_{0}-E_{min}\right),\;x_{e}\in <0,1>,$$

where $E_{min}$ is the value of Young’s modulus of void elements, $E_{0}$ represents Young’s modulus of the AW2011 alloy, and *p* is a penalization factor, the value of which depends on the Poisson’s ratio. The value of the penalization factor *p* = 3 was chosen according to the relation described in the paper by Bendsøe and Sigmund [28]. To ensure solvability of the equation, a small value of the Young’s modulus had to be considered for the void elements; in our analysis, the value was set to $E_{min}=10^{-6}$ MPa.

For the purposes of the study, the objective of TOP was to minimize the compliance problem. Due to the character of our problem (multiple load-cases), the solution was divided into multiple objectives, which are summed up as

(8) $$\min:c\left(x_{e}\right)=\sum_{j=1}^{J}\sum_{e=1}^{n}E_{e_{\left(x_{e}\right)}}{\left\{u_{j,e}\right\}}^{T}\left[k_{0}\right]\left\{u_{j,e}\right\},$$

where $c(x_{e})$ is the deformation energy, $\left\{u_{j,e}\right\}$ is the displacement vector of the element of the *j*-th load case, and [*k*${}_{0}$] is a stiffness matrix of each planar element with Young’s modulus equal to one. The volume constraint was defined using the equation

(9) $${\left\{x_{e}\right\}}^{T}\left\{v_{e}\right\}-f\cdot \sum_{e=1}^{n}\left\{v_{e}\right\}\leq 0,$$

where *f* is the volume fraction and $\left\{v_{e}\right\}$ represents the array with element volumes. The design variable xe ranges between 0 and 1.

TOP comes with inherent (deep-rooted) problems such as the checkerboard pattern problem [29], and, hence, it is appropriate to use a filtering method. The filtering method causes the density of elements $x_{e}$ to depend on the neighboring elements [30]. However, this method causes the formation of areas with intermediate density, also known as grey areas, and it is, therefore, necessary to choose the density limit value $x_{lim}$ above which the elements will be preserved (while, below it, they will be removed). In this case, a density filter was used. The density filter is averaging three variables from the element’s neighborhood (i.e., the derivation of the objective function, derivation of the constraint function, and density). The information was used to automate the above process in MATLAB. The script is capable of multi-load step 2D topology optimization after entering the input data. After performing the TOP, the resulting shape was analyzed and smoothed in Stage IV.

### 2.4. Stage IV: Analysis and Smoothing of the Calculated Discrete Shape

The result of the topology optimization is a map determining the importance of the elements. The map distinguishes elements to be preserved and to be removed. After removing elements, the geometry model contained sharp edges resulting from the finite element discretization. If the final design of the cam was made according to the discrete model map, it would contain places with high stress concentration. For this reason, it was advisable to smoothen the calculated discrete shape and, therefore, to remove these stress concentrations.

The 2D Non-Uniform Rational Basis Splines (NURBS) were used for the purposes of the shape approximation. NURBS are implemented in many Computer Aided Design (CAD) software solutions for their excellent modeling capabilities [31]. NURBS facilitates the construction of conic sections, basic bodies (cylinder, cone, etc.) [32]. A point belonging to the NURBS is defined as

(10) $$P^{new}\left(q\right)=\frac{\sum_{i=0}^{l}w_{i}P_{i}M_{i,k}\left(q\right)}{\sum_{i=0}^{l}w_{i}M_{i,k}\left(q\right)},$$

where $w_{i}$ is a vector of weights for the control points (which are, in our case, nodes from finite element mesh), $P_{i}$ are their Cartesian coordinates, and $M_{i,k}$ is the NURBS blending function of *k*-th degree, which is defined by means of the Bernstein’s polynomials. The number of points ranged from *i* to *l*. The curves generally do not pass through control points, they approximate them. The blending function is assigned to the individual control points. Without the weights, the points belonging to the curve can be obtained as linear combinations of coordinates of control points and of the blending functions. By changing the position of the control point, it is possible to change the shape of the curve locally. To obtain a smoother shape, it was advantageous to use the highest degree of the polynomial, which depends on the number of control points. In this work, the *k* values were automatically calculated individually for each curve, ranging from 5 to 250.

### 2.5. Stage V: Modeling Load-Bearing Capacity

**Figure 7:**
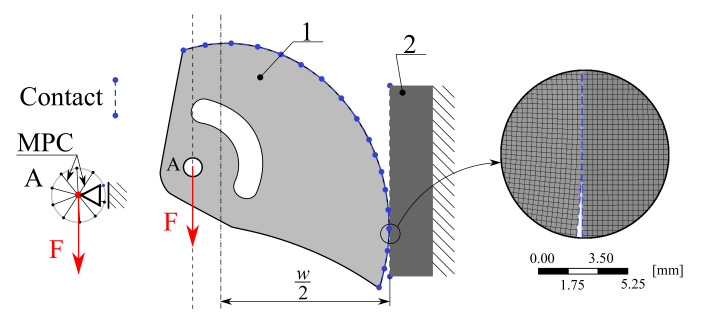
Boundary conditions for the nonlinear computational model (1—scheme of the cam, 2—jaw) and detail of mesh.

To find out if the smoothed discrete shape was optimal, it was necessary to analyze the load-bearing capacity of the cam. A full nonlinear computational model was created in AWB to capture the physical behavior during testing; this model also included nonlinear material behavior, large deflections, and contact behavior. In this case, however, the computational model was complemented by a planar geometrical model of the jaw, see Figure 7.

The model was designed as parametric to simulate real testing conditions according to the standard EN 12276 [6]. The angle of the cam rotation is the only parameter in this model so it is possible to analyze its behavior within the entire working range. The Augmented Lagrange Contact Algorithm was used for modeling the contact between the cam and the jaws; the coefficient of static friction was set, as discussed in Section 2.1, to $f_{T}$ = 0.27. The jaw was fixed on the right edge, all degrees of freedom (two displacement values) were set to 0 mm. The pin was replaced with the Multi-point constraint (MPC algorithm) with a rigid behavior formulation. The Support A represents a symmetric boundary condition for the pilot node of the MPC. A gradually increasing force was applied on the pilot node, up to the value of $F_{max}$ = 3.50 kN corresponding to 14.0 kN for the whole SLCD.

An elasto-plastic material model of AW 2011 with kinematic hardening was used for the cam, an elastic isotropic material model of S235 for the jaw. The material constants are presented in Table 2 and Table 3. A planar element with quadratic displacement approximation (PLANE183) was used to describe the behavior of the cam and jaw. The size of the edge of one element $e_{size}$ = 0.30 mm was defined. A statistics of the mesh is presented in Table 7.

## 3. Results

Based on the presented materials and methods, the cam was optimized using updates of the geometric model. The following sections present the results obtained in Stages III, IV, and V.

### 3.1. Topology Optimization

**Figure 8:**
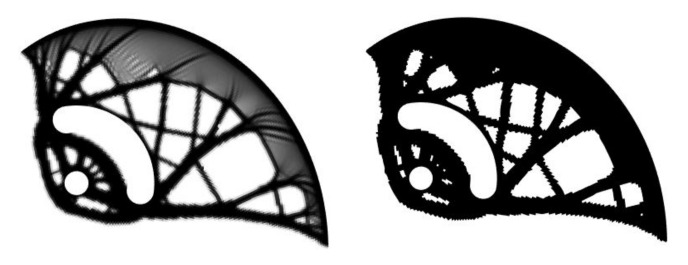
The first optimization run–density distribution field in grey-scale: black color—keep element, white color—remove element (**left**) and pseudo-density distribution field using the density limit value $x_{lim}=$ 0.1 (**right**).

When using a linear computational model for topology optimization, the value of the volume fraction was set to *f* = 50%. The first calculated cam design (density distribution field) was obtained in the first optimization run, see Figure 8 (a gray-scale field in the left). To determine the final shape, a density limit (cut-off value for preserving/removing the elements) had to be set. For the purposes of the work, the density limit value was set to $x_{lim}=$ 0.1, at which no checkerboard field pattern arose. Figure 8 also presents the pseudo-density distribution field (the two-color black and white field in right). As can be seen from the figure, the weight reduction was insufficient.

**Figure 9:**
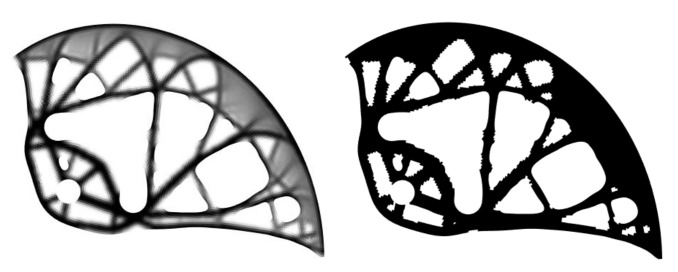
The second optimization run–density distribution field in grey-scale: black color—keep element, white color—remove element (**left**) and pseudo-density distribution field using the density limit value $x_{lim}=$ 0.1 (**right**).

Therefore, the initial geometric model was updated using the first calculated cam design, see Figure 4. This means that, after the first optimization run, the pseudo-density distribution field was smoothened and used as a new (updated) geometric model for input into Stages II–IV. Subsequently, a new finite element mesh was generated for the next optimization run with the same parameters. This simple loop is suitable for calculating the sufficient weight reduction while maintaining a constant volume fraction of *f* = 50%. Figure 9 shows the density distribution field from the second optimization run. This shape was, after smoothing, considered optimal.

### 3.2. Analyzing and Smoothing of the Calculated Discrete Shape

**Figure 10:**
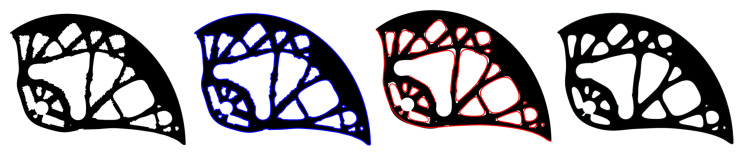
Four steps of the smoothing process (from the left): (i) importing the calculated shape, (ii) analyzing the external shape, (iii) approximation of external nodes, and (iv) generation of the smoothed geometric model.

Before the analysis and smoothing of the calculated shape, zero-density elements were removed from the pseudo-density distribution field. The subsequent smoothing process was divided into four steps for each optimization run, see Figure 10: (i) importing the calculated shape (the mesh), (ii) analysis of the external shape (finding external nodes and edge sorting), (iii) approximation of external nodes by the curve of the selected degree, and (iv) generation of the smoothed geometric model in the Standard triangle language (STL) file format. The above-mentioned procedures were created and automated in MATLAB using 2D NURBS.

**Figure 11:**
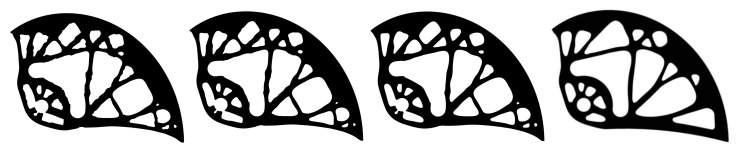
Cam shape after smoothing achieved by calculations using various values of polynomial degrees of *k* = 5, 10, and 100 (from the left), and the optimal cam design with *k* = 250 (right).

Figure 11 presents the smoothing geometric models for three different degrees of approximation, namely for *k* = 5, 10 and 100. To obtain a smoother shape, it was advantageous to use the highest degree of the polynomial, which depends on the number of control points. In this work, the *k* values were automatically calculated individually for each curve, ranging from 5 to 250. After smoothing, it was necessary to manually modify the smoothed geometric model to preserve the pin holes. Furthermore, some holes were ignored and others merged together. These modifications were made manually by the authors, taking into account the limitations of the manufacturing process. The optimal cam design is depicted in Figure 11 on the right.

### 3.3. Modeling of Load-Bearing Capacity

**Figure 12:**
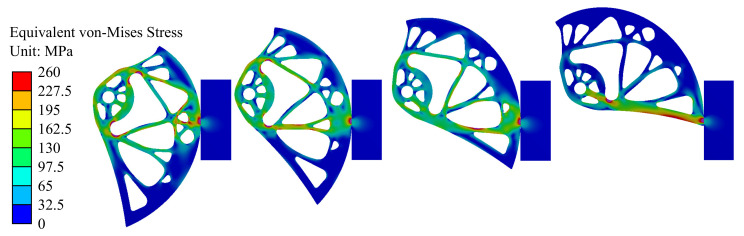
Von Mises stress fields corresponding to the force $F_{min}$ = 5.00 kN (*F* = 1.25 kN) for different widths of the rock crack *w* = 75, 86, 97, 108 mm (from left).

**Figure 13:**
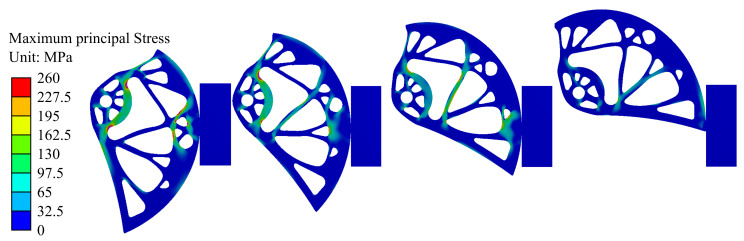
Maximum principal stress fields corresponding to the force $F_{min}$ = 5.00 kN (*F* = 1.25 kN) for different widths of the rock crack *w* = 75, 86, 97, 108 mm (from left).

Modeling of load-bearing capacity was performed for nine positions of the cam within the working range. Two moments were analyzed. The first one was the moment when the force applied on the whole device reached the $F_{min}$ = 5.00 kN (*F* = 1.25 kN per cam). Figure 12 shows the Von Mises stress fields for five different cam rotations corresponding to the widths of the rock crack *w* = 75, 86, 97, 108 mm (from the left). In Figure 12, the red color shows places exceeding the yield stress. Figure 13 shows the maximum principal stress fields for the same cam rotations. The figure presents critical areas with the highest values of tensile stress, i.e., areas that are most susceptible for developing cracks.

The second moment was the one when the plastic strain reached the limit value $\varepsilon_{pl}$ = 5.50%. The load-bearing capacity for that moment was determined for each position. The final load-bearing capacity was determined on the basis of the minimum value corresponding to $F_{max}$ = 7.09 kN for the maximal width of the rock. A data summary is presented in Table 8. It is necessary to note that the nonlinear buckling effect did not occur in either of the defined moments.

## 4. Discussion

In this paper, we presented the process of determining the optimal cam design of the spring-loaded camming device (SLCD) using topology optimization. The suggested methods offer benefits in the way of weight reduction while maintaining the stiffness and are sufficient from the point of view of the EN 12276 standard. The design procedure was divided into five stages.

The virtual modeling of the cam by FEM using a linear computational model and its boundary conditions was performed in Stage II. It should be mentioned that simplifications were used to reduce the computational complexity of the problem. The most notable of these was the conversion of a spatial problem into a planar one. On the other hand, the results from the solution of such a planar problem offer considerable advantages during production on CNC machines. Should the problem be solved as a 3D optimization problem, other production methods, such as 3D printing, would be preferable. Nevertheless, additive manufacturing, although offering freedom in designing complex geometries, is unsuitable for cam printing due to the high price of the product and due to possible formation of pores during printing, which can negatively affect the mechanical properties of the material [33]. In Stage III, topology optimization of the linear computational model was performed using procedures prepared in MATLAB (which were previously validated using AWB) [11]. Results could be possibly further improved through solving the problem as nonlinear with large displacements or through the introduction of another objective function of topology optimization (e.g., the optimization of natural frequencies). In the last stage, the load-bearing capacity was modeled using a nonlinear computational model. Elasto-plastic material model behavior was considered, which facilitated obtaining information about the plasticity regions. The greatest plastic deformation occurred in the area of contact where a more accurate friction computational model should be used. To obtain simulation results of higher quality, it would be necessary to use a 3D model of the cam; another way would be to perform material tests and refine the parameters of the material model. To verify the functionality, it would be necessary to test it according to the standard EN 12276. A tensile test of the AW2011 material on a special device would likely support an even more accurate calculation of the load-bearing capacity presented in the study [34].

This solution needed to be tested in terms of its functionality and standard. At present, two testing methods are available. The first one is the experimental measurement using a tensile device, which is expensive as it would require the production of the test piece and a customized setting for measurement. It is also more time-consuming. However, that method is accurate and is legally required if the product is should be placed on the market. On the other hand, the numerical (computational) method is cheaper and provides results with accuracy sufficient for the purposes of this study and optimization.

When modeling the load-bearing capacity of the cam, the pin connection is modeled using MPC. To increase the accuracy of the results in the area of the pin, it is necessary to use the cam-pin connection model by means of a friction contact. The computer modeling of bolted connections in a similar industrial problem is described in conference proceedings [35] by Horyl and Marsalek.

Linear elements were used for the construction of the linear model, which was optimized. The main advantage of the linear elements was the simplification of contact conditions (jaws contact was replaced by joint prescribed for one node in the contact). Quadratic elements were used in the model with two contact bodies, which was solved in AWB. The main advantage of quadratic elements was contact description.

The load in the strength analysis is considered static, which is based on the standard. Nevertheless, in the case of a climber’s fall, a dynamic effect arises in the rope, which would be transferred to the cams. The size of the shock itself is most dependent on the weight of the climber and the depth of the fall. In addition, the direction of the forces could change, which would affect the contact pair. These phenomena should be studied and included in further analyses. The impact test modeling method is described by Horyl et al. [36], where the authors describe the modeling of the dynamic process influencing the attachment of the seat to the wagon frame in the case of crash.

The original material of the PB6’s cam was considered in the new design process; in future research, it would be appropriate to also consider other materials. Saga et al. [37] described impact toughness testing of a composite material, which could be a promising material for the cams as well. The potential advantages of the composite material include the possibility to design the orientation of the fibers with respect to the loading of the part, thus improving the toughness and potentially reduced weight of the cams. When working on this issue in the future, it would be also interesting to apply the same optimization method to an entire set of cams with various cam sizes, possibly also on single-axle SLCDs.

## 5. Conclusions

This paper describes a method for preparing an optimal design for a cam of a dual-axle spring-loaded camming device (SLCD). Due to the device adaptability to a rock crack, a large number of load cases can arise, which would be difficult to simulate using existing MATLAB procedures. If the cam was optimized only for the load cases described by the standard, it would be unusable in other possible positions. For this reason, a script was designed to solve the boundary conditions within the entire contact curve. The loading force was considered to be parallel to the ideal wall, but, in real use, this occurs rarely. Simulating that would, however, increase the number of boundary conditions and, in effect, the computational time.

Due to the assumed simplification of the problem (i.e., the use of 2D linear elements), the problem can be solved directly using FEM by obtaining an inverse stiffness matrix. In the case of a larger number of degrees of freedom, another method for solving the equation would be probably more appropriate. Makropoulos et al. [38] discussed solving elasticity (and mainly elastoplasticity) problems while using parallel solvers.

Ole et al. [25] recommended using a filtering technique for preventing the occurrence of checkerboard patterns. A density filter, which offers a relatively robust design, has proven to be a very advantageous filtering method. Its disadvantage is the occurrence of grey zones; to obtain a black and white solution, it is necessary to set a density limit, which is difficult to determine without the previous experience of the solver. To obtain the final shape, the FE model must be further modified. The adjustment consists of removing sharp edges using smooth curves (2D) or surfaces (3D). NURBS, which are used in computer graphics, proved to be a suitable tool. The use of the maximum possible degree of the polynomial, which depends on the number of control points, has proven to be very advantageous. This simple procedure could be replaced by merging the topology optimization and NURBS into one solver. The paper by Giulio Costa et al. [39] described topology optimization combined with NURBS hypersurfaces. The need for user intervention into the designed geometry to ensure the preservation of the functionality of the device (in our case, the preservation of pin holes) is a small disadvantage of the chosen procedure.

**Figure 14:**
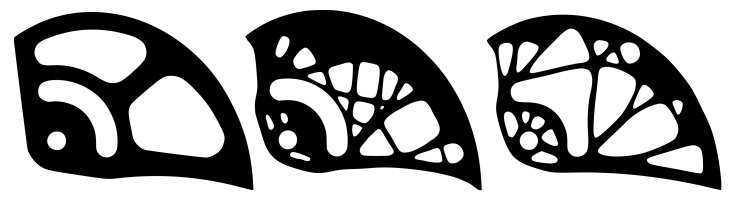
PB6’s cam (**left**), first optimization run (**middle**), optimal cam design (**right**).

A planar model of a single cam was used to verify the geometry. The boundary conditions were chosen to correspond to the real form of testing given by the standard. The simplified model brings the advantage of speedy calculation. The results of the computational model indicate that the optimal cam design would meet the standard. A visual comparison of the PB6’s cam with the first optimization run and optimal cam design is presented in Figure 14.

In this paper, topological optimization was utilized for the material distribution of the cam. The presented solution reduces the weight of the cam by 14%, with a resulting cam weight of 28.9 g, see Table 9. As each device contains four cams, the total weight reduction per device is 19.6 g, which, considering that the set of SLCDs is needed during a usual climb, adds up to a significant value. Moreover, it should be noted that the PB6 SLCD is already a result of a long experimental development and, as such, it has been already empirically optimized. However, as far as production time (and, in effect, costs) are concerned, the new cam design brings a significant increase in the price of the solution.

Table 9 also compares the load-bearing capacity of the optimal cam design with that of the BP6 cam. From the perspective of load-bearing capacity, the optimal cam design meets the requirements defined by the EN 12276 standard, although the load-bearing capacity is lower than that of the PB6 cam. This is caused by the assumptions taken for the solution, namely the definition of the force *F* = 5.00 kN, the density limit value $x_{lim}$ = 0.1, volume fraction *f* = 50%, and other numerical parameters.

It is necessary to note that the weight reduction is not effective in terms of the ratio between the weight and load-bearing capacity. However, the authors focused on meeting the standard EN 12276, which defines the minimum load capacity $F_{max}$ = 5.00 kN. Should new requirements be defined for the device, the shape of the cam can be easily modified by including new assumptions and prescription of other numerical parameters. EN 12276 prescribes only two positions for testing the load-bearing capacity (25% and 75% of the range width). Nevertheless, as indicated by our results (see Table 8, the values of load-bearing capacity achieved in other positions are lower, and it might be worth considering whether testing in the extreme positions would be beneficial as well.

The performance of the new shape of the cams must be tested in extreme conditions taking into account the impact loads acting in different directions. In this paper, the authors consider only the ideal conditions described in Section 1.3.

Finally, an additional benefit of the cam solution is the attractive design of the cam resulting from the presented algorithms, which could contribute towards the commercial success of the presented device.

The computing time for a linear model including the topology optimization was 2.03 s. per one load case (267 load-cases in total) on a standard machine (workstation Intel i5-8600, 6 cores, 16 GB RAM, 500 GB SSD). Approximately 0.21 s was needed for each iteration in the individual load-case. The full nonlinear model used for load-bearing capacity testing required much more time: the mean computing time for each cam position was 74.9 min (summary 9 positions, total time 674 min).

## Figures and Tables

**Table 1:** Mean value of the individual ratios of working range for selected types of PRG.

Active PRG	Ratio of the Working Range	Passive PRG	Ratio of the Working Range
Dual-Axle SLCD	*R* = 41%	Tricam	*R* = 37%
Single-Axle SLCD	*R* = 35%	Hexentrics	*R* = 26%
LTMS	*R* = 22%	Nuts	*R* = 10%

**Table 2:** Material constants of AW2011 alloy.

Property	Symbol	Value	Unit
Poisson ratio	μ	0.33	-
Young’s modulus	*E*	72.5	GPa
Yield stress	$\sigma_{y}$	260	MPa
Ultimate stress	$\sigma_{u}$	370	MPa
Elongation	*A*	0.06	-
Tangent modulus	$E_{T}$	2 343	MPa
Limit plastic strain	$\varepsilon_{pl}$	0.055	-
Density	ρ	2 820	kg·m ${}^{-3}$

**Table 3:** Material constants of S235 steel.

Property	Symbol	Value	Unit
Poisson ratio	μ	0.30	-
Young’s modulus	*E*	206	GPa

**Table 4:** Coefficient of friction between AW2011 and S235.

Material	Material	Coefficient of Static Friction	Limit Angle of the Beam
AW2011	S235	$f_{T}$ = 0.27	α = 15.1${}^{\circ }$

**Table 5:** Parameters representing the initial geometric model of the cam.

Parameter	Symbol	Value	Unit
Initial radius of the cam	$r_{0}$	35.7	mm
Coefficient of static friction	$f_{T}$	0.27	-
Maximal radius of the cam	$r_{max}$	65.0	mm
Maximal rotation of the cam	$\varphi_{max}$	127	${}^{\circ }$
Diameter of the hole for pin	ϕd	6.00	mm
Distance between pins	*e*	16.0	mm
Thickness of the cam	*t*	6.00	mm

**Table 6:** Linear computation model mesh statistics.

Nodes	Elements	DOF
17,800	17,500	35,600

**Table 7:** Nonlinear computation model mesh statistics.

Nodes	Elements	DOF
121,000	39,000	242,000

**Table 8:** Load-bearing capacity for different rotations of cam.

Width of the Rock Crack *w* [mm]	Plastic Strain $\varepsilon_{\mathrm{pl}}$ [%] for Force $F_{\min}$ = 5.00 kN	Load-Bearing Capacity $F_{\max}$ [kN] for Limit Plastic Strain $\varepsilon_{\mathrm{pl}}$ = 5.50%
64.0	2.74	7.33
69.5	2.75	7.34
75.0	2.49	7.83
80.5	2.21	8.26
86.0	1.97	8.98
91.5	1.87	8.13
97.0	1.76	9.88
102.5	3.02	10.21
108.0	0.90	7.09

**Table 9:** Comparison of weights and load-bearing capacities.

Type	Weight of the Cam	Load-Bearing Capacity
PB6’s cam	$m_{c}$ = 33.8 g	$F_{max}$ = 14.0 kN
Optimal cam design	$m_{c}$ = 28.9 g (14.5%)	$F_{max}$ = 7.09 kN (49.3%)
EN 12276 limit	-	$F_{max}$ = 5.00 kN

## Data Availability

Data sharing is not applicable to this article.

## Abbreviations

The following abbreviations are used in this manuscript:

AWB	Ansys Workbench
SLCD	Spring-Loaded Camming Device
FE	Finite Element
FEM	Finite Element Method
NURBS	Non-Uniform Rational Basis Splines
PRG	Protection Gears
LTMS	Lever-Type Mechanical Stopper
TOP	Topology Optimization
SCDM	SpaceClaim Direct Modeler
2D	Two-dimensional
3D	Three-dimensional
SIMP	Solid Isotropic Material with Penalty
DOF	Degrees of Freedom
CAD	Computed Aided Design
STL	Standart Triangle Language
CNC	Computer Numerical Control
MPC	Multiple Point constraints
PB6	Permon Biaxial 6
